# Our Paris Correspondence

**Published:** 1860-03

**Authors:** Robert Battey

**Affiliations:** Paris


Vol. V.] MARCH, 1860. [No. 7.

### ORIGINAL COMMUNICATIONS.

### ARTICLE I.

### *Our Paris Correspondence.*

Paris, January, 1860.

Messrs. Editors:—I have not forgotten the promise made you on the eve of my leaving home, to write you of my observations and experiences abroad. I write now, not so much with the view of making good the pledge, as for the purpose of reminding you that an American medical man in Paris, desiring to avail himself of a tithe of the privileges here afforded him for instruction, has not time to *read* the journals, much less is he able to write for them. In support of this position, I have but to refer you to your own experience of a student's life in Paris. You will well recollect that the daily solicitude is—not to find profitable employment for one's time—but rather to find time for all the advantages we desire to secure.

There are but few medical novelties engaging the public attention here just now. The several Societies are discussing, more or less, the subject of "Hypnotism," or magnetic somnambulism. This peculiar anæsthetic condition, evidently identical in all its essential features with the mesmeric influence, which pervaded the entire civilized world some years ago, is now brought about by more simple and rational means, the apparatus used for the purpose being merely a bright metallic ball upon the end of a rod, which is attached —by means of an adjustable wire frame—to an elastic band, passing around the head. This ball is adjusted to a position over the bridge of the nose distant say 1½ to 2 inches from either eye. The patient's attention is now fixed upon the ball steadily for 20 minutes, in which time the hypnotic influence should make its appearance. The anæsthesia, when produced, is asserted to be quite as complete as that by ether or chloroform, and admits of the performance of the most painful operations without a consciousness of pain on the part of the patient.

The range of its applicability appears not to be very extensive, since no adult male has yet succumbed to its influence, and the successful application in women and children is perhaps rather the exception than the rule. That it is often applied successfully, seems to be well established. I saw a trial made of it some weeks since in the Hotel Dieu, in the female ward of M. Jobert (de Lamalle). After waiting for 30 minutes upon the hynotizer, M. Jobert threw it down in a pet, and fell back upon his chloroform.

There is a curious observation which has been developed in the investigation of this subject, viz: its effect upon animals. It is substantiated, that if a common fowl be held in the hands, and a chalk line drawn down the centre of the beak, the fowl, after being kept quiet for some minutes, becomes completely hypnotized, and is wholly insensible to pain—may be pricked with a pin or cut with a knife with perfect impunity. This experiment is said to succeed more uniformly, if, in addition to the line upon the beak, a line be also drawn upon the floor and the beak of the fowl held upon it.

There has heretofore been much uncertainty in the mensurations of the lower extremities, for the purpose of determining the shortening which may result from fracture or dislocation of the femur, growing out of the fact that the position of the pelvic planes at an exact right angle with the central line of the body, is of difficult attainment, and is, at the same time, essential to a just comparison of the two limbs. Mons. Dasheta has recently given us a means of determining with almost mathematical exactitude, the precise amount of deviation in the unsound limb. He proceeds upon the assumption, (which is, for practical purposes, true) that a line drawn from the anterior superior spinous process of the illium to the centre of the tuberosity of the ischium will bisect the acetabulum, and this line he takes for the base of a triangle, the other two sides of which are determined by measuring the distance from the inner condyle of the femur, first to the anterior superior spinous process, and then to the tuberosity. These measurements are made upon both limbs, and by comparing the two triangles thus obtained, upon established geometrical principles, he arrives at the absolute difference in length of the two limbs.

In the limited space and time allowed me I cannot enter into his reasoning, or show clearly his results, but if the subject shall prove of interest to yourselves and readers, I may at a future day be induced to give you the entire demonstration and result.

M. Nelaton gives a very interesting course of clinical lectures three times a week, in connection with his morning round at the Faculty Hospital, opposite L'Ecole de Medicine. In connection with this course, he presented, a few days since, a case from his private practice, in which, after the removal of a tumor above the inner aspect of the elbow joint, and the necessary ligation of an arterial branch quite close to the main trunk of the brachial artery, he placed a precautionary loose ligature upon the trunk just above the origin of the branch. Time passed on—hemorrhage occurred—the precautionary thread was tightened—supposing the hemorrhage to proceed from the branch, the primary ligature was examined, but proved to be quite firm. At a subsequent visit this came away, and with it a section of quite three-quarters of an inch in length of the brachial artery.

The section was placed under the microscope by M. Robert and ascertained to be true arterial tissue in a gangrenous state. M. Nelaton attributes this unusual occurrence to the artery having been denuded in the removal of the tumor which rested immediately upon it. A similar result from a a like cause had occurred to M. Velpeau, at La Charite, some time ago.

Nelaton—apparently 50—is in the prime of his physical and intellectual vigor. He speaks distinctly, with ease and animation, and always draws a full house. Upon one of his clinic days, not long since, he essayed the performance of Bozeman's operation for Vesico Vaginal Fistula—worked for two hours, as I am informed, very industriously in the location of three points of suture, and left the case finally in a condition which he acknowledged to be quite unsatisfactory. A cure was not anticipated, but what the result was I have not yet learned.

M. Nelaton is unquestionably a superior operative surgeon —perhaps has no more successful rival in Paris—but seems, like all Parisian surgeons, to encounter special difficulties in this essentially American operation.

The Salle Acouchment of the Faculty, under the charge of M. Paul Dubois and his adjunct, M. Pajoe, is now attended chiefly by the latter, who also delivers a tri-weekly course of clinical lěctures. This course is numerously attended, and is of a highly interesting and practical character.

M. Pajoe doubtless considers himself a young man—we would rank him in America in middle life, say 40—and evidently rates his intellectual powers at their full value. For this he is not blamable, since he is truly a man of vigorous and cultivated mind; besides, this consciousness of his strength gives him firmness and composure at the bed-side, with ease and fluency in the lecture-hall—qualities so commendable that one may well overlook a little occasional manifestation of vanity. Pajoe is fine looking—one might almost call him handsome—and is a lively, fluent and impressive lecturer—articulates forcibly and distinctly, and is a favorite with Americans.

The acouchment ticket for this ward is accessible to all, and entitles the holder to the privilege, in his turn, of the "toucher," and the management of labor. Here, also, the student has ocular demonstration of the process of parturition upon the living subject, a privilege of doubtful utility and nowhere else obtainable.

Some days since, the "Sage-Femme" of the Salle Acconchment was called upon to perform the functions of her office in a carriage at the gate of the Hospital—an occurrence which, I am told is not unfrequent, as the institution does not receive the patients until the pains of labor set in.

I probably hazard nothing in saying that M. Velpeau, at La Charite, still maintains his position at the head of the list of French surgeons, notwithstanding his unpleasant connection with the notorious "Medecin Noir," (black doctor,) as he is called here. He is evidently in the evening of life, and cannot for very many years more, wield the surgical knife. His voice is weak, and among the large numbers who crowd around him at the bed-side and in the lecture room, very few derive any substantial advantage from his teachings. Some weeks ago, a stranger offered him some new surgical device. M. V. very promptly referred him to the Academy, and quickly turned his back. His experiences with the *Noir* have evidently left a tender cicatrix behind.

Of the trial, conviction and punishment (very light, was it not?) of the cunning charlatan, Vrier, for his unauthorized exercise of the divine art of healing, I need not speak; you, doubtless, have it detailed and commented upon in all your exchanges.

At the Hotel Dieu, M. Jobert (de Lamballe) has charge of three wards, two male and one female. On the left of the door as we enter the female ward, stands a charcoal furnace, glowing with its pent-up caloric of highest intensity, and supporting upon its hearth the handles of certain potential instruments, whose clubbed extremities nestled in the fiery bath of ignited carbon, imbibe to saturation the fervent heat.

We pass through the little operating room, where stands the cushioned table, and other appropriate paraphernalia, into the Salle, to await the coming of the Emperor's favorite surgeon. The garcon is busily engaged in putting an extra polish upon the old oaken floor, and with an eager eye searches out the smallest stray dossil of lint that might perchance catch the quicker eye of his fault-finding master. Another stands at the bell-rope, ready to give the note of warning upon the first audible sound of the haughty tread of *the* surgeon of the Hotel Dieu. M. Jobert enters—a man of middle age—formerly handsome, still good looking—with an air of haughtiness in his carriage one seldom meets with. He seems to bid defiance to all laws of gravitation in carrying his centre of gravity behind the base upon which he stands. His internes and externes offer a respectful salutation, but he seems as insensible to all such polite attacks upon his dignity as a majestic iceberg. His followers at the bed-side are scanty and meek, and receive but few favors at his hands, unless it be in the subjection and rigid discipline of his medical staff and attendants. If you chance, momentarily, to obstruct his light, he demands in a stern tone, "Get out of my *day!*" and lectures you soundly for your imprudence. Woe be to the unlucky interne who gets in his light or jostles his elbow.

M. Jobert has under his care the great mass of the cases of vesico-vaginal fistula occurring in Paris. He discards in to-to the American improvements, and operates upon a method of his own. In the chronic inflammations and ulcerations of the cervix uteri he employs the actual cautery—his "bouton de fer"—and apparently with results equally happy and much more prompt than we obtain in America from the pencil of nitrate of silver. The tinner's furnace and soldering irons are certainly, in appearance at least, frightfully formidable weapons to take into the chamber of a delicate female, yet I am assured and fully believe that the pain occasioned by the cautery is not more than by the nitrate, and in the great majority of instances no pain whatever is experienced. M. Jobert, as he sits at his operating table and growls and cuts and scolds, does not usually make a very favorable impression upon strangers, but it must be conceded that he is an expert operator in general surgery.

M. Robert, who also has charge of three surgical wards at Hotel Dieu, makes less noise and draws larger audiences. He has not the air of a man of much industry and spirit. The few clinical lectures I have heard from him were dull and uninteresting.

M. Trousseau, of the Medical Department of the same hospital, draws large audiences at the bed-side and in the lecture room. He is a middle-aged man, of fine personal appearance, and a model gentleman—easy and graceful in his manners. I need not say that he is a man of talent and mental culture—his fame is not confined to Europe. As a lecturer, he is peculiarly happy in his style and address, and justly stands at the head of the Faculty in this important qualification. He has recently given a series of highly interesting lectures upon diphtheritic paralysis, which have been reported in extenso in the "Gazette des Hopitaux."

Laraboisiere is so distant from the Latin quarter, where students usually lodge, that few of them attend it. Mons. Chassaignac—the leading spirit in this institution—however, always draws a good audience upon his clinic days—every Monday. In all its appointments for the care of the sick, this splendid institution is the best in Paris, and perhaps is not equalled in the world.

M. Chassaignac has three wards—two male and one female. There seems to be a discrimination exercised at the central bureau in the allotment of patients to the several hospitals. I have already remarked that the ward of M. Jobert is rich in cases of V. V. Fistula; the champion of the Ecrasseur, on the other hand, is furnished, to a great extent, with cases particularly demanding the employment of that instrument, and the drainage tubes. At this clinic we are struck with the very large proportion of cases involving operations upon the rectum—as, for instance, fistula, hemorrhoids and carcinoma, and also hypertrophy and tumors of the cervix uteri, and cancer of the tongue. These patients are deemed fit subjects for the Ecrasseur. He has, too, a large number of cases of diffuse abscess for the drainage tubes.

Last week a woman was presented who had been operated upon by ecrasment two years ago for a carcinoma of the recto-vaginal septum. She was relieved for the time, and suffered little inconvenience and pain for the space of fourteen months, when she returned with a renewal of the cancerous tumor, and was again relieved. She now applied for a third ecrasment, which was performed, and I saw her on yesterday doing well, the wound in apparently healthy granulating condition. Considering the unpromising character of this class of cases, the operative procedures of M. C. appear quite successful.

At this Hospital the use of the plaster bandage is very general in fractures, and also in the treatment of the "tumor blanche." A roller bandage is first applied, and then thickly smeared over with a cream of Plaster-of-Paris, which forms a firm, immovable case for the limb. This apparatus is, of course, applied after all inflammation has subsided.

M. C. treats fractures of the clavicle by encasing the forearm and arm as high as the middle of the humerus in this plaster case. He then, with several thicknesses of the roller, forms a tight sling, suspending the arm from the opposite shoulder so as to raise the lame shoulder as high as possible. This sling is also smeared with the plaster, so as to form a complete, firm horse-collar, (excuse the expression,) which is immovably joined to the cased arm. No auxiliary pad is employed. He believes the sole indication to be, to raise as high as possible the shoulder upon the side of the fracture, and keep the parts at rest, and argues that this apparatus secures a more perfect freedom from motion, while it does not press upon any limited surface to chafe and excoriate, but distributes the pressure so widely as greatly to promote the comfort of the patient. His cases, so far as I have been able to judge, do perfectly well.

M. Ricord, of the Hopital du Midi, has for some time been recreating, and his wards have been under the charge of his chief interne. It was currently reported that he had reached the age at which surgeons are expected to retire from the public service, but he is now back at his post again and making his daily rounds. I was surprised to hear from an eminent Parisian surgeon, the caustic remark—" M. Ricord is but a syphilitic fungus grafted upon the Hospital Midi."

At the meeting of the Chirurgical Society, last week, the venerable surgeon of Le Charite was present, and took part in the discussion. I observed also MM. Robert and Chassaignac among the members in attendance. An interesting case of extrophy of the bladder was exhibited in an adult male peasant from the Provinces.

The Academy of Medicine have now under consideration " The Artificial Illumination of the Cavities of the Body by the Aid of Luminous Tubes." It is the device of two Germans—" MM. Th. du Moncel et Ruhmkorff,"—and consists of an electric light which may, by means of tubes, be transmitted into the recesses of the mouth and throat, vagina or rectum. The practical application of it in the vaginal tube was exhibited last week in the service of M. Becquerell, at La Pitie, who was pleased with it, and expressed much satisfaction with the increased facility it affords in these examinations. M. Cl. Bernard is upon the commission having the matter in charge, and will probably soon report the result of their investigations to the Academy, when we shall have more light upon the subject.

The American Medical Society holds its weekly meetings for the reception of reports from the various Hospitals, and discussion of medical topics. For two months past the supply of journals from America has entirely ceased, from some unknown cause, so we are left entirely in the dark as regards American medicine.

Of the American medical men sojourning here for professional improvement, there were, at one time, twelve from our State—Georgia—a larger number than from any other State in the Union. We number now nearly or quite ten—two have recently left for home, and three or four more will return during the spring and summer.

Respectfully, yours, ROBERT BATTEY.

